# Balancing stromal-induced complexity in 3D ovarian cancer models through heterotypic co-culture with fibroblasts or architected micro-scaffolds

**DOI:** 10.1186/s13036-026-00670-9

**Published:** 2026-03-30

**Authors:** Eglė Žymantaitė, Karolina Limanovskaja, Linas Jonušauskas, Vita Pašukonienė, Neringa Dobrovolskienė, Agata Mlynska

**Affiliations:** 1 Laboratory of Immunology, National Cancer Institute, P. Baublio g. 3B, Vilnius, LT-08406 Lithuania; 2 Institute of Biosciences, Life Sciences Center, Vilnius University, Sauletekio 7, Vilnius, LT-10257 Lithuania; 3 Vital3D Technologies, Saulėtekio al. 15, Vilnius, LT-10224 Lithuania; 4 Department of Chemistry and Bioengineering, Vilnius Gediminas Technical University, Sauletekio al. 11, Vilnius, LT-10223 Lithuania

**Keywords:** 3D cultures, Tumor heterogeneity, Heterotypic cell cultures, Scaffolds, 3D printing, 2PP

## Abstract

Tumor heterogeneity limits the reproducibility and interpretability of in vitro ovarian cancer models. In this study, we established a comparative framework that intentionally modulates stromal-induced complexity through two complementary approaches: scaffold-free stromal co-culture with patient-derived cancer-associated fibroblasts (CAFs) or fibroblast cell line WS1, and scaffold-based micro-scaffolds printed using two-photon polymerization (2PP) with poly(ethylene glycol) diacrylate (PEGDA). We analyzed four ovarian cancer cell lines (A2780, SKOV3, COV362 and OV7,) stemness/epithelial-mesenchymal transition (EMT), and TGF-β. We then evaluated their 3D spheroid formation and growth dynamics across different seeding densities. In ultra-low-attachment plates, SKOV3, COV362, and OV7 formed round, compact spheroids across different seeding densities, while A2780 produced loose aggregates. Co-culturing with early-passage CAFs or WS1 fibroblast cell line (in 2:1, 1:1, and 1:2 ratios of cancer cells : fibroblast) rescued A2780‘s ability to form spheroids and increased the compactness of OV7 and COV362 in a line- and ratio-dependent manner in co-culture with CAFs. Compared to 2D cultures, 3D spheroids exhibited higher expression of stemness/EMT and angiogenesis related genes. In contrast, the PEGDA scaffolds (with pore sizes of 65, 100, and 130 µm) standardized early attachment and surface coverage for both spheroid-competent line SKOV3, and a line that does not typically form spheroids, A2780. These scaffolds also shifted EMT/stemness gene expression without elevating vascular endothelial growth factor (VEGF) levels compared to scaffold-free 3D cultures. Together, these results provide practical guidance: utilize fibroblast co-culture to study the stromal-induced complexity and cell-to-cell interactions, including the rescue of cell lines that typically do not form spheroids, and employ printed micro-scaffolds to control 3D culture variability.

**Supplementary information:**

The online version contains supplementary material available at 10.1186/s13036-026-00670-9.

## Introduction

Tumor heterogeneity, which includes variations in morphology, transcriptional profiles, metabolism, and metastatic potential, significantly impacts cancer progression and treatment effectiveness. This complexity, found both within a single tumor (intratumoral heterogeneity) and among tumors from different patients (intertumoral heterogeneity), poses a major challenge in oncology research and clinical practice, often resulting in therapeutic failure and drug resistance [1–3]. The intricate interactions of diverse cellular populations and their dynamic relationships with the tumor microenvironment are not well represented in preclinical 2D in vitro models [4, 5]. Recently, 3D culture models, such as organoids and organotypic spheroids, composed of cancer cell lines, have emerged as more accurate representations of tumor biology in vitro. These 3D models address the limitations of traditional 2D cell cultures and better capture the inherent biological complexity and heterogeneity of tumors [6, 7].

Generally, 3D culture techniques can be divided into scaffold-free and scaffold-based strategies. Scaffold-free methods, like ultra-low-attachment plates (both flat and U-bottom), hanging drop techniques, microwell systems, and agitated bioreactors, encourage self-assembly via cell-to-cell adhesion, enabling the rapid formation of size-controlled spheroids. Advanced techniques such as magnetic levitation and droplet microfluidics enhance this approach for high-throughput and uniform 3D cultures [8, 9]. In contrast, scaffold-based methods involve embedding or seeding cells within natural hydrogels or synthetic polymers, which allow for controlled stiffness and degradability. 3D-printed scaffolds can also introduce defined pore or strut geometries, standardizing architecture independently from stromal components [10, 11]. Across different techniques used, incorporating co-cultures with stromal cells (fibroblasts, cancer patient-derived fibroblasts (CAFs)) can yield heterotypic cultures that better represent tumor–microenvironment crosstalk. Fibroblasts frequently form the spheroid core, with seeding order and cancer cell : fibroblast ratio determining compactness, architecture and proliferation. These co-cultures reproduce in vivo-like core–shell organization and hypoxic/necrotic core [12]. Despite the range of available 3D methodologies, most studies prioritize generating spheroids with minimal characterization for preclinical research rather than explicitly addressing heterogeneity. The MISpheroID Consortium created a knowledge base that includes 3,058 spheroid experiments. Their findings revealed that the methodological setups used in these experiments are highly varied and frequently lack detailed descriptions. Both empirical evaluations and interlaboratory comparisons have shown that even minor differences in formation methods, medium compositions, or size selection can significantly affect spheroid metrics [13]. The huge variety of spheroid generation techniques developed till today makes it difficult to compare results across studies [13, 14]. Direct, side-by-side comparisons of stromal co-culture and engineered micro-architectural 3D scaffolds are relatively uncommon, limiting practical guidance for choosing the right techniques to enhance or constrain heterogeneity for specific experimental goals.

In this study, we established a comparative in vitro framework that models and intentionally modulates tumor heterogeneity in ovarian cancer cell lines by employing two practical approaches: patient-derived fibroblast and immortalized fibroblast cell line, co-cultured in a scaffold-free system, and two-photon polymerization (2PP)-printed poly(ethylene glycol) diacrylate (PEGDA) micro-scaffolds with varying pore sizes, in a scaffold-based system. We test the hypotheses that fibroblasts can rescue spheroid formation and modulate compactness in a ratio- and line-dependent manner, while PEGDA scaffolds can standardize 3D culture formation. Our side-by-side design aims to provide practical guidance on when to leverage stromal crosstalk to enhance heterogeneity versus when to utilize engineered architecture to control variability in 3D cultures. Early-passage ovarian CAFs (P2) or the fibroblast cell line WS1, co-seeded at ratios of 1:1, 2:1, and 1:2 (cancer cell : fibroblast), improved spheroid formation in the A2780 cell line and enhanced spheroid compactness. Meanwhile, PEGDA scaffolds with defined pore sizes (65, 100, and 130 µm) provided consistent architectural constraints under otherwise similar conditions. Our methods align with growing evidence that CAF-tumor co-cultures enhance spheroid formation in ovarian cancer models [12]. Additionally, 2PP allows for architecturally precise PEGDA-based scaffolds that are well-suited for robust 3D culture formation [10].

## Materials and methods

### Cell culture and reagents

The SKOV3 human ovarian cancer cell line and the human fibroblast cell line WS1 were obtained from American Type Culture Collection (ATCC®, cat no. HTB77™; cat no. CRL-1502 ™). Human ovarian cancer cell lines A2780, COV362, and OV7 were purchased from the European Collection of Authenticated Cell Cultures (ECACC, cat no. 93112517; cat no. 07071904; cat no. 96020764). A2780 cells were cultured in RPMI 1640 medium (Gibco™, cat. no. 11875093) supplemented with 10% fetal bovine serum (FBS; Gibco™, cat. no. 16140071) and 1% penicillin–streptomycin (PS; Gibco™, cat. no. 15140122). SKOV3 and COV362 cells were maintained in Dulbecco’s Modified Eagle Medium (DMEM; Gibco™, cat. no. 11,965,092) supplemented with 10% FBS and 1% PS. OV7 cells were cultured in DMEM/F-12 medium (Gibco™, cat. no. 11320033) supplemented with 10% FBS, 1% PS, 0.5 µg/mL hydrocortisone (Sigma-Aldrich, cat no. H0888), and 10 µg/mL insulin (Sigma-Aldrich, cat no. I6634). The WS1 fibroblast cell line was cultured in ATCC-formulated Eagle’s Minimum Essential Medium (ATCC®, cat. no. 30–200) supplemented with 10% FBS. All cell lines were maintained at 37 °C in a humidified atmosphere of 5% CO₂ and passaged upon reaching ~80% confluence. Cells were used for experiments between passages 3 and 20. Cell viability was routinely assessed by Trypan Blue exclusion (Gibco™, cat. no. 15250061).

### Spheroid formation

Spheroid formation was evaluated by seeding A2780, COV362, SKOV3, and OV7 in UltraPure™ agarose (Invitrogen™, cat. no. 16500500) covered U-bottom 96-well plates, prepared following protocol [15]. Cells were seeded at densities ranging from 3000 to 7000 cells per well, with 200 µL of cell line preferred cell culture media as described above. The 3D cell cultures were maintained at 37 °C in a humidified atmosphere with 5% CO2 for 13 days. Cell cultures were observed and imaged every three days using an inverted phase-contrast microscope (OPTIKA ITALI IM-5). Image analysis was performed using AnaSP (v2.0) software, as described in [16, 17]. AnaSP calculates: Volume, number of voxels of the volume of the foreground, reconstructed using the ReViSP algorithm [17]; Diameter, diameter of a circle with the same area as the foreground, computed as √(4 × Area/π); Sphericity, as 4 × π × Area/Perimeter, with “Area = area of the original mask” and “Perimeter = perimeter of the original mask”.; and Compactness as 4 × π × Area/Perimeter^2^, with “Area = area of the original mask” and “Perimeter = perimeter of the original mask”.

### Spheroid migration assay

One spheroid was transferred into each well of a 24-well plate and imaged at time points 0 h, 6 h and 24 h using an inverted microscope. The area invaded by each spheroid at these time points was quantified using Fiji (ImageJ) software.

### Isolation of cancer-associated fibroblasts from ovarian tumors

Cancer-associated fibroblasts were isolated from ovarian tumor tissue obtained from female patient (*N* = 1) diagnosed with stage III serous ovarian carcinoma undergoing surgical resection at the Department of Oncogynecology, National Cancer Institute, Vilnius, Lithuania. Patient provided written informed consent prior to surgery. The study was approved by the Vilnius Regional Biomedical Research Ethics Committee (permit no. 2022/9–1456–925) and conducted in accordance with institutional guidelines, regulations, and the principles of the Declaration of Helsinki. Fresh tumor specimens were collected intraoperatively and immediately transported to the Laboratory of Immunology at the National Cancer Institute in sterile 1× phosphate-buffered saline (PBS; Gibco™, cat. no. 10010023) supplemented with 2% penicillin-streptomycin (PS; Gibco™, cat. no. 15140122). Upon arrival, tissue samples were rinsed with PBS containing 2% PS, finely minced into 1–3 mm^3^ fragments, and enzymatically digested in collagenase type II (1 mg/mL; Sigma-Aldrich, cat. no. SCI103) at 37 °C for 30 minutes with gentle agitation. Following digestion, the cell suspension was filtered, centrifuged, and resuspended in growth medium consisting of Dulbecco’s Modified Eagle Medium (DMEM; Gibco™, cat. no. 11965092) supplemented with 20% fetal bovine serum (FBS), and 1× PS. Cells were plated in 25 cm^2^ culture flasks and maintained at 37 °C in a humidified incubator with 5% CO₂. CAF populations were enriched using differential trypsinization and expanded for two passages. Cells were then harvested and cryopreserved in freezing medium (90% FBS, 10% dimethyl sulfoxide (DMSO)) and stored at − 80 °C for short term storage.

### Heterotypic spheroid formation

Heterotypic spheroids were generated by co-culturing 5000 cells per well, consisting of ovarian cancer cell lines (A2780, COV362, SKOV3, OV7) and cancer-associated fibroblasts at ratios of 1:1, 2:1, and 1:2 (cancer cells : fibroblasts), or by co-culturing the A2780 and SKOV3 cell lines with the fibroblast cell line WS1. Cells were seeded in low-attachment 96-well round-bottom plates to promote spheroid formation, prepared following protocol described above. 3D cultures were maintained at 37 °C in a humidified atmosphere with 5% CO₂ for 6 days. Spheroid morphology and growth were observed and imaged every 3 days using an inverted microscope, and image analysis was performed using AnaSP (v2.0) software [16, 17] as described above.

### Fabrication of 3D scaffolds

One pre-polymer, poly(ethylene glycol) diacrylate (PEGDA, Mn 700; Sigma-Aldrich, Burlington, MA, USA cat no. 455008), mixed with 1% (w/v) of the photoinitiator 2-Hydroxy-4’-(2-hydroxyethyl)-2-methylpropiophenone (Irgacure 2959; Sigma-Aldrich cat no. 410896), was used to print the 3D scaffolds following the protocol previously described in [10]. Architecture of scaffold followed general spherical shape, with ends being slightly flattened for better adhesion to the glass slide it was printed on. Internal architecture was periodic gyroid, with pore sizes of 65 µm, 100 µm and 130 µm. Pore sizes were defined as the biggest cross-section of any opening within the structure. Additional bent tube-like features were imbedded in the scaffold to facilitate liquid circulation in the structure. Finally, hatching step of 5 µm was chosen, so final structure would be porous and would allow materials to diffuse through the structure.

### Cell seeding on printed scaffolds

PEGDA scaffolds were sterilized by immersion in 70% ethanol, followed by exposure to ultraviolet (UV) light for 20–30 minutes. After sterilization, the scaffolds were carefully detached, washed with PBS, and placed into the wells of a 96-well plate, prepared as described above. Each scaffold was submerged in cell culture medium, depending on the cell line, for 1 hour prior to cell seeding.

Cells were seeded at a density of 14,000 cells per well in 200 μL of supplemented medium, as this seeding density was optimized in a previous study [10]. A2780 and SKOV3 ovarian cancer cell lines were cultured at 37 °C in a humidified atmosphere with 5% CO₂ for 6 days. As a control, 3D cell cultures without scaffolds were prepared following the same protocol, with 14,000 cells seeded per well. The development of 3D cultures was monitored daily using an inverted microscope.

### Flow cytometry

Flow cytometry was performed to characterize the expression of cell surface markers in the ovarian cancer cell lines A2780, COV362, SKOV3, and OV7, as well as characterize cancer-associated fibroblasts. Cancer cells were incubated for 20 minutes at 4 °C with the following monoclonal antibodies: ESA (CD326)-PE (Miltenyi Biotec, MACS, clone HEA-125, cat. no. 130–113–826, 1:50), CD44-APC (Miltenyi Biotec, MACS, clone DB105, cat. no. 130–113–893, 1:50), CD90-PE/Cy7 (BioLegend, clone 5E10, cat. no. 328123 1:25), CD73-PE (BioLegend, clone AD2, cat. no. 344003 1:25), CD105-APC (BioLegend, clone: 43A3, cat. no. 323207 1:25), and CD155-Pacific Blue (BD Bioscience, clone: SKII0.4 and cat. no. 748241 1:50). CAFs were characterized separately using monoclonal anti-human antibodies: CD90-PE/Cy7, CD73-PE, and CD105-APC. Cells were stained using the recommended antibody concentrations according to manufacturer instructions. Following incubation, cells were washed twice with CellWASH buffer (BD Biosciences, cat. no. 349524) and resuspended in CellWASH for acquisition. Flow cytometry data were collected using a BD LSR II flow cytometer (BD Biosciences) and analyzed with BD FACSDiva software (v6.2; BD Biosciences) and FlowJo software (v10.9; BD Biosciences). Unstained cells were used as negative controls. A minimum of 20,000 events was collected for each sample.

### Evaluation of gene expression by real-time qPCR

Total RNA was extracted from cell culture samples on day 6 using TRIzol™ Reagent (Invitrogen™, cat. no. 15596018) in combination with the RNeasy Mini Kit (Qiagen, cat. no. 74104), following the manufacturers’ protocols. The concentration and purity of the extracted RNA were assessed using a NanoDrop™ 2000 spectrophotometer (Thermo Scientific™). Absorbance was measured at 260 nm and 280 nm, and RNA concentration was calculated accordingly. The A260/A280 ratio was used to evaluate RNA purity, with values between 1.8 and 2.0 considered acceptable. RNA samples were stored at − 80 °C until further use. For cDNA synthesis, 300 ng of total RNA from each sample was reverse transcribed using the Maxima First Strand cDNA Synthesis Kit (Thermo Fisher Scientific, cat. no. K1642), according to the supplied instructions. Quantitative PCR was performed in triplicate using the Azure Cielo™ 3 Real-Time PCR System. Each 10 μL reaction contained 5 μL of Maxima SYBR Green qPCR Master Mix 2X (Thermo Fisher Scientific, cat. no. K0241), 2.5 μL of 0.8 μmol/L sequence-specific forward and reverse primer mix (Supplementary Table 1), 1 μL of cDNA, and 1.5 μL of nuclease-free water. Thermal cycling conditions were as follows: an initial denaturation at 95 °C for 5 minutes, followed by 40 cycles of 10 seconds at 95 °C (denaturation) and 30 seconds at 60 °C (annealing/extension).

Gene expression levels were normalized to the Ribosomal Protein L13a gene (*RPL13A*). The experiment was repeated twice. Data analysis was performed using Azure Cielo™ 3 Real-Time PCR System software (v1.0.0.300).

### Statistical analysis

Data normality was verified with the Shapiro–Wilk test and equality of variances with Levene’s test. Unpaired Student’s t-test was used for pairwise comparison. Statistical analysis was performed using R (v4.3.1), *p*-values < 0.05 were considered statistically significant.

## Results

**Fig. 1:**
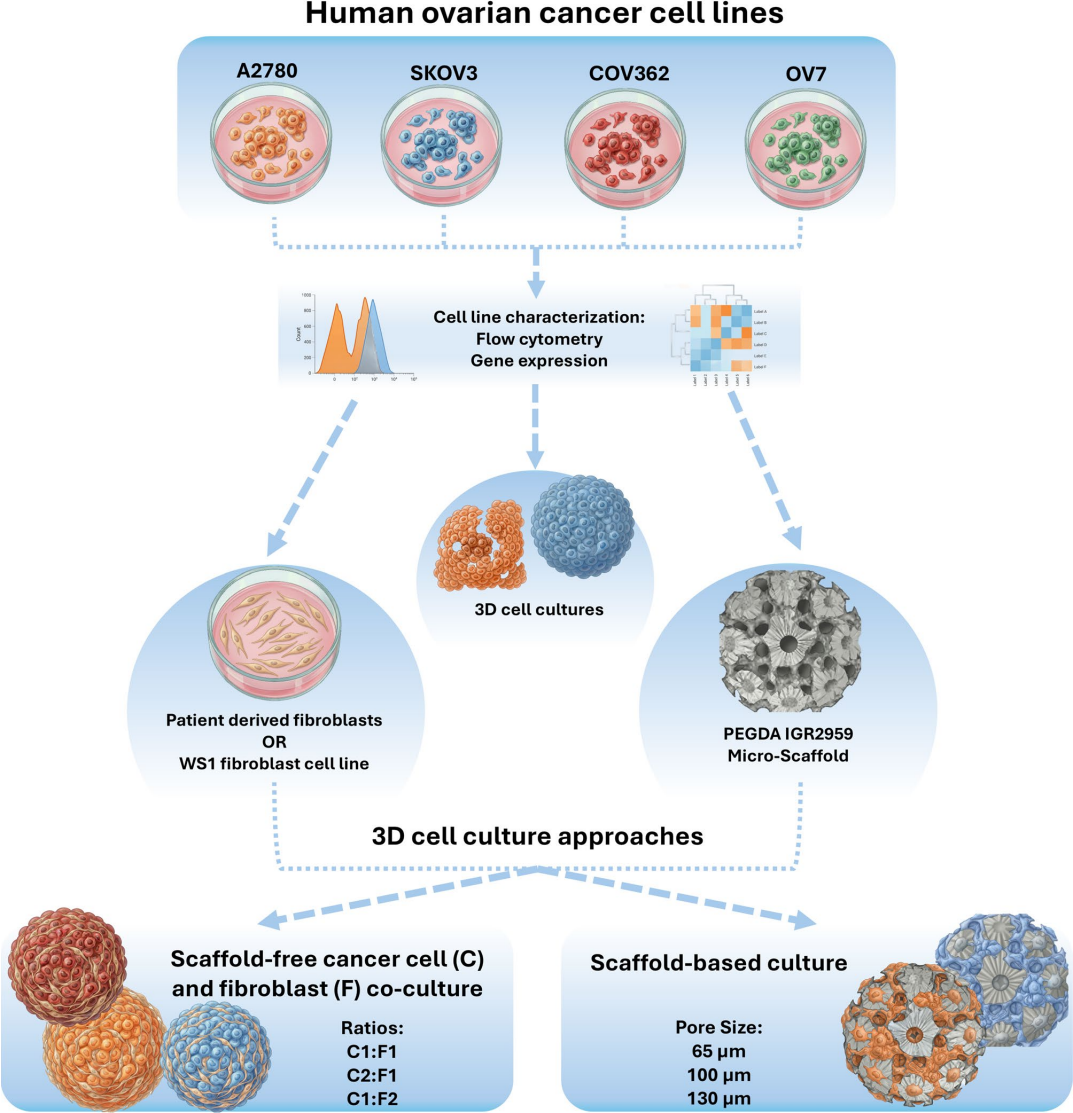
Schematic summary of the study design

In this study, we used a comparative in vitro framework to model and deliberately modulate tumor heterogeneity in ovarian cancer cell lines using two complementary approaches: scaffold-free tumor–fibroblast co-culture spheroids incorporating CAFs or an immortalized fibroblast cell line, and a scaffold-based system using two-photon polymerization (2PP)-printed poly (ethylene glycol) diacrylate (PEGDA) micro-scaffolds with defined pore sizes. A schematic overview of the study design is shown in Fig. 1.

### Characterization of ovarian cancer cell lines

Four ovarian cancer cell lines: OV7, COV362, SKOV3, and A2780, were analyzed by flow cytometry to assess markers associated with epithelial cell properties, cell adhesion, immune modulation, and cancer stemness. The epithelial markers measured were ESA (epithelial specific antigen, also known as EPCAM) [18] and CD44, a glycoprotein involved in adhesion, migration, and often linked to cancer stemness [19]. Additional markers included CD90 (also known as THY1), associated with tumourigenicity [20]; CD73 (also known as NT5E), an enzyme contributing to immune suppression in the tumor microenvironment [21]; CD155 (also known as PVR), an immune checkpoint molecule described in tumor immune evasion and cell–cell interactions [22]; and CD105 (also known as ENG), a glycoprotein related to angiogenesis and tumor progression [23].

**Fig. 2:**
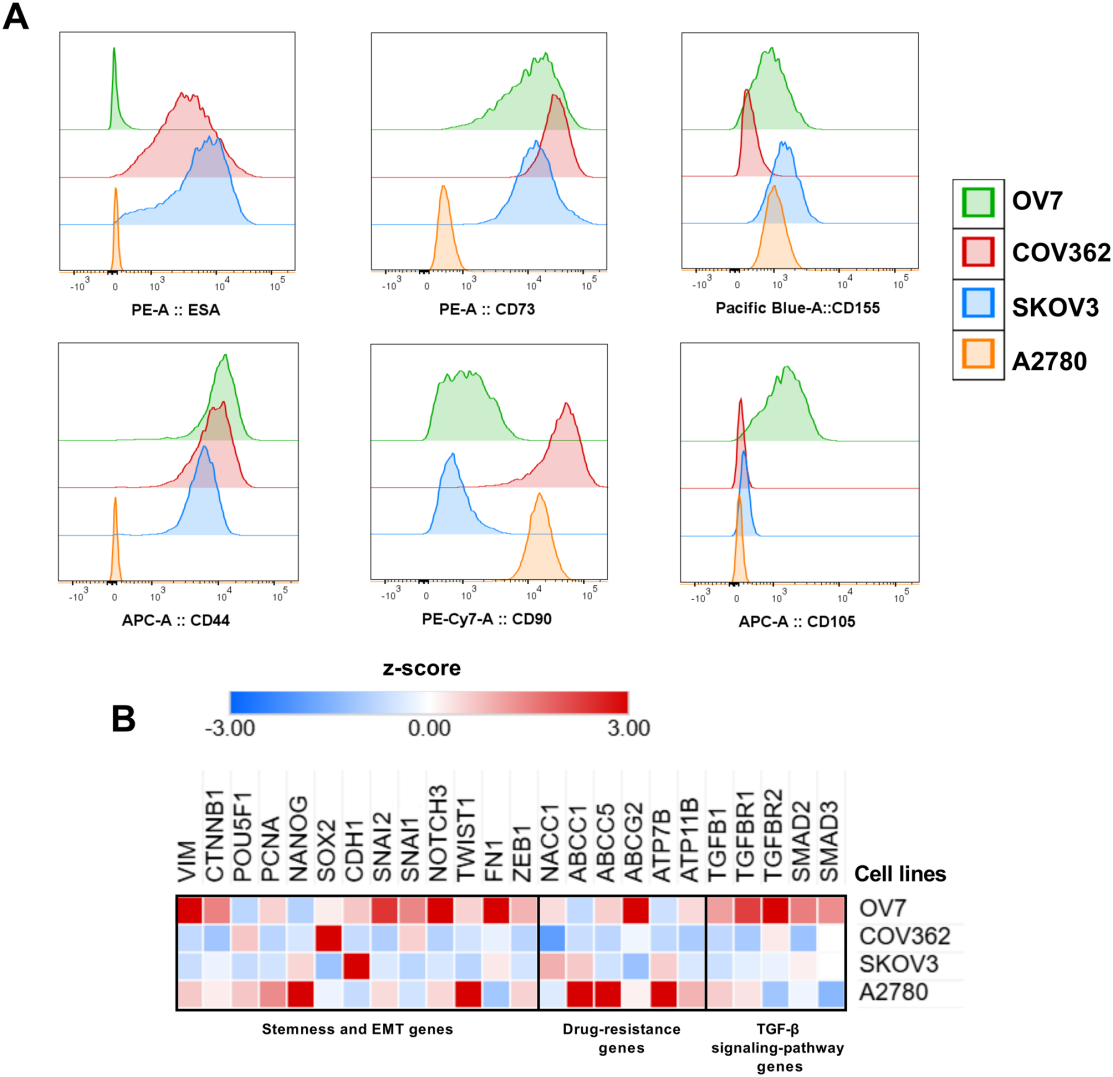
Phenotypic and genetic profiling of four ovarian cancer cell lines. (**A**) flow cytometry analysis of OV7, COV362, SKOV3, and A2780 cell lines. The markers measured were ESA (epithelial specific antigen, EPCAM), CD44, CD90 (THY1), CD73 (NT5E), CD155 (PVR), and CD105 (ENG). (**B**) real-time PCR analysis of genes related to stemness and epithelial-mesenchymal transition (EMT), drug resistance, and TGF-β signaling. Values are per-gene z-scores

Flow cytometry revealed clear phenotypic differences among the cell lines (Fig. 2A). OV7 cells showed strong expression of CD44, CD73, CD155, and CD105, with low levels of CD90 and no detectable ESA expression. COV362 cells had high expression of ESA, CD73, and CD44, and the highest CD90 expression among the four lines, but displayed very low CD155 and no CD105 expression. SKOV3 cells expressed ESA, CD73, CD155, and CD44, with slightly lower CD90 levels, and no CD105 expression. In contrast, A2780 cells showed low expression of CD73 and CD155, and no detectable ESA, CD44, or CD105 expression, but high expression of CD90.

Gene expression profiling by real-time PCR further revealed distinct gene expression patterns among the four ovarian cancer cell lines for genes related to stemness and epithelial-mesenchymal transition (EMT), drug resistance, and TGF-β signaling (Fig. 2B). In ovarian cancer, TGF-β signaling regulates EMT and tumor–stroma crosstalk, driving cellular plasticity and metastatic progression [24]. Accordingly, TGF-β pathway genes were included in the baseline gene-expression panel to evaluate cell line–specific differences associated with 3D growth states. Within the stemness and EMT-associated genes, OV7 cells showed the highest expression, compared to the other cell lines, of *VIM, CTNNB1, SNAIL1, SNAIL2, NOTCH3*, and *FN1*. COV362 displayed elevated levels of *POU5F1, SOX2*, and *SNAIL1*. SKOV3 cells expressed high levels of *NANOG*, *CDH1*, and *FN1*, whereas A2780 exhibited the highest expression of *NANOG, PCNA*, and *TWIST1*. For drug-resistance genes, OV7 cells had elevated expression of *NACC1, ABCC5*, and *ABCG2,* while COV362 did not show high expression of any drug-resistance gene relative to the other cell lines. SKOV3 displayed increased expression of *NACC1, ABCC1*, and *ATP7B,* whereas A2780 showed high expression of *ABCC1*, *ABCC5*, and *ATP7B*. In the TGF-β signaling pathway genes, OV7 expressed high levels of all analyzed genes. COV362 showed increased expression of *TGFBR2,* SKOV3 displayed elevated *SMAD2*, and A2780 exhibited higher expression of *TGFB1* and *TGFBR1*.

Taken together, these cell marker and gene expression profiles highlight distinct phenotypic and molecular characteristics among the four ovarian cancer cell lines, which may contribute to their abilities to form and maintain 3D cultures.

### Establishment and optimization of ovarian cancer spheroids

**Fig. 3:**
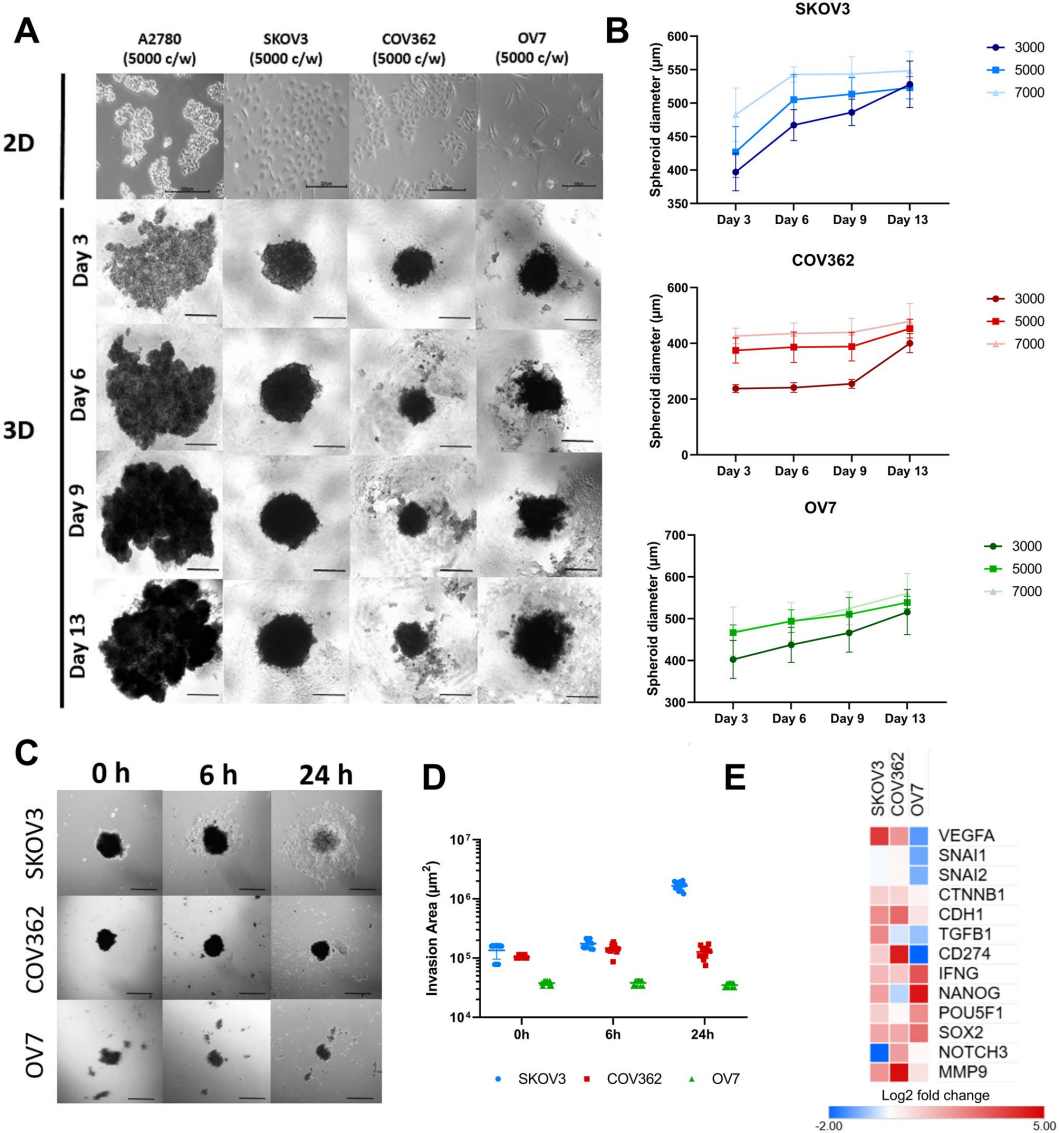
Spheroid formation and characterization using different ovarian cancer cell lines. (**A**) Representative images of SKOV3, COV362, and OV7 spheroids cultured at the optimal seeding density of 5,000 cells/well (c/w) between day 3 and day 13. The A2780 cell line formed only small, loose aggregates. Scale bar represents 300 μm. Magnification 40x (**B**) Spheroid growth kinetics measured as spheroid diameter over 13 days at seeding densities ranging from 3,000 to 7,000 cells/well (c/w). Data are presented as mean ± SD, *n* = 8, *N* = 3 biological replicates. (**C**) Migration assay of 13-day-old SKOV3, COV362, and OV7 spheroids (5,000 cells/well) transferred onto 24-well adherent plates and imaged at 0 h, 6 h, and 24 h. Migration area was quantified relative to initial spheroid area. Scale bar represents 200 μm, magnification x40, *n* = 13, *N* = 3 biological replicates. (**D**) Quantitative analysis of invasion area measured from spheroid core to furthest migrated cells presented as (µm^2^). (**E**) Heatmap of genes upregulated or downregulated in 3D spheroids compared to 2D monolayer cultures. Genes include angiogenesis-related (*VEGF*), epithelial–mesenchymal transition (EMT)-associated (*SNAI1, SNAI2, CTNNB1, CDH1, TGFB1*), immune-related (*CD274, IFNG*), cancer stemness-associated (*NANOG, POU5F1, SOX2*), aggressiveness/chemoresistance-related (N*OTCH3*), and immune system remodeling *(MMP9*) genes. Values are presented as log2 Fold change

The ability of four human ovarian cancer cell lines (A2780, SKOV3, COV362, and OV7), which differ in their molecular profiles as described above, to form reproducible 3D spheroids in vitro was assessed. Cells were seeded in ultra-low attachment plates at densities ranging from 3,000 to 7,000 cells per well and were cultured for 13 days (Fig. 3A). Spheroid formation was evaluated by tracking growth dynamics and morphological changes over time. Within the first 24 hours, all cell lines migrated towards the center of the well, forming a uniform cell mass. By day 3, SKOV3, COV362, and OV7 had developed round, compact spheroid structures with characteristic morphology. In contrast, A2780 failed to form 3D spheroids; instead, only loose cell aggregates were observed. Based on these results, only SKOV3, COV362, and OV7 3D cultures were further characterized (Fig. 3A).

To determine optimal seeding density for spheroid formation, SKOV3, COV362, and OV7 were cultured at 3,000, 5,000, and 7,000 cells per well. Spheroids were imaged every three days, and their diameters were measured (Fig. 3B). All three lines formed spheroids across the tested seeding densities. Growth curves confirmed spheroid viability and showed that cultures grew until reaching a stable size under the given conditions. Based on growth dynamics, 5,000 cells per well was identified as the optimal seeding density for all three lines. On day 13, mean spheroid diameters (mean ± SD) were: SKOV3 (523.0 ± 16.0 μm), COV362 (450.8 ± 33.7 μm), and OV7 (537.9 ± 14.8 μm).

The migratory capacity of spheroid-forming cell lines was next assessed. Spheroids generated from 5,000 cells and cultured for 13 days were transferred to adherent 24-well plates and imaged at 0 h, 6 h, and 24 h (Fig. 3C). SKOV3 spheroids attached within 1 h; by 6 h, a cell layer had formed, and cells began migrating away from the spheroid. At 24 h, the spheroid appeared more transparent with extensive cell dispersal, and by 48 h, it had fully dissociated into a monolayer. COV362 spheroids attached only after 24 h and showed no strongly visible migration. OV7 spheroids mostly disintegrated during transfer, failing to maintain compactness, and those that remained intact did not attach. Migration capacity was quantified by measuring the migration area from the spheroid core (Fig. 3D).

Finally, gene expression profiles of SKOV3, COV362, and OV7 were compared between 2D monolayer cultures and 3D spheroids (5,000 cells/well, 13-day culture) (Fig. 3E). Across all three lines, genes associated with angiogenesis, epithelial-mesenchymal transition (EMT), cancer stemness, and immune system remodeling were upregulated in 3D spheroids relative to 2D cultures. *VEGF* gene expression was upregulated in SKOV3 and COV362 spheroids, suggesting enhanced angiogenic potential. EMT-related genes *CTNNB1* (β-catenin) and *CDH1* (E-cadherin) were increased in SKOV3, COV362, and OV7 spheroids, consistent with the formation of compact 3D spheroid cell cultures. Stemness-associated genes *NANOG, POU5F1*, and *SOX2* were also upregulated in all three lines in 3D culture, indicating that spheroid conditions promote cancer stem-like characteristics.

### Patient-derived CAFs enhance spheroid formation in ovarian cancer cell lines

Fibroblasts were isolated from ovarian tumor tissue obtained during surgery from patient diagnosed with stage III serous ovarian cancer. The fibroblast cultures were characterized at passage 2 by flow cytometry using three fibroblast-associated markers: CD105, CD90, and CD73 [25], all of which were strongly expressed (Supplementary Fig. S1A). To assess their spheroid-forming ability, fibroblasts were seeded in low attachment plates at a density of 5,000 cells/well and cultured under 3D cell culturing conditions. By day 3, fibroblasts formed dense, round spheroids, which became more compact by day 6. Under 2D conditions fibroblasts retained a characteristic flat, elongated morphology (Supplementary Fig. S1B).

**Fig. 4:**
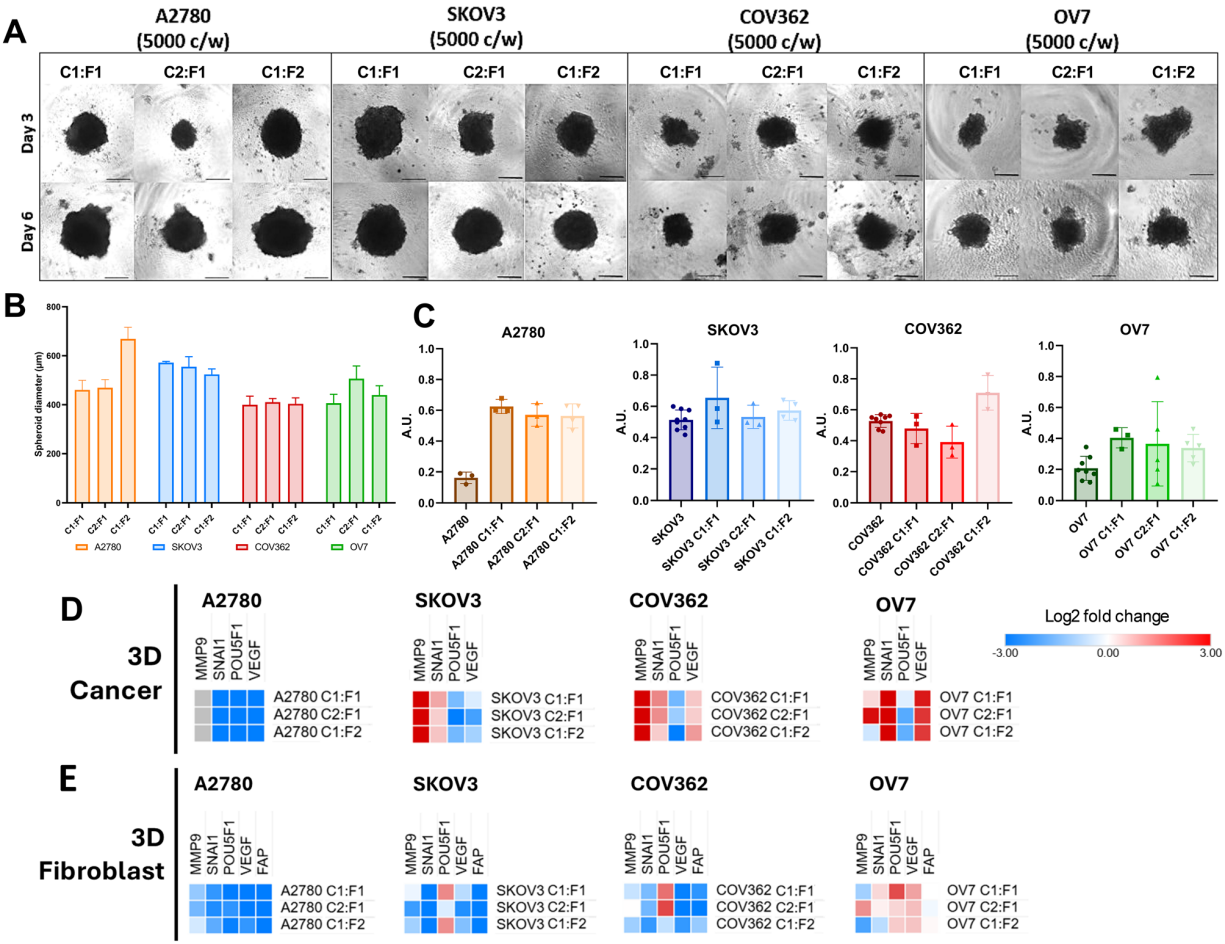
Fibroblast co-culture modulates spheroid formation, size, compactness and gene expression in ovarian cancer cell lines. (**A**) Representative bright-field images of A2780, SKOV3, COV362 and OV7 3D cultures co-cultured with patient-derived fibroblasts at cancer : fibroblast ratios C1:F1 (1:1), C2:F1 (2:1) and C1:F2 (1:2) (total 5,000 cells/well (c/w)). Images are shown at day 3 and day 6. Scale bar represents 300 μm, magnification 40×. (**B**) Spheroid diameter at day 6. Bars show mean ± SD. *n* = 2–6, *N* = 1 biological replicates. (**C**) Spheroid compactness at day 6. A2780 shows low compactness in monoculture but increases at all co-culture ratios (C1:F1, C2:F1, C1:F2). Bars represent mean ± SD; *n* = 2–6, *N* = 1 biological replicates; points represent individual spheroids. (**D**) Heatmaps show log2 fold-change for *MMP9, SNAI1, POU5F1, VEGF* normalized to each line’s 3D cancer monoculture. (**E**) Heatmaps show log2 fold-change for *MMP9, SNAI1, POU5F1, VEGF, FAP* normalized to 3D fibroblast monoculture

At passage 2, after characterization, fibroblasts were co-cultured with the previously tested ovarian cancer cell lines A2780, SKOV3, COV362, and OV7 (Fig. 4A). Co-cultures were prepared at cell-to-fibroblast ratios of 1:1 (C1:F1), 2:1 (C2:F1) and 1:2 (C1:F2), with 5,000 total cells per well, and grown for 6 days. By day 6, all spheroids had reached their optimal size for analysis. In monoculture, A2780 cells did not form compact spheroids, only cell aggregates; however, when co-cultured with patient-derived fibroblasts, they developed well-defined, stable spheroids by day 3. SKOV3, COV362, and OV7 formed spheroids under all tested conditions; however, co-culture with fibroblasts generally enhanced the compactness and structural stability of spheroids in the COV362 and OV7 cell lines.

Spheroid diameters were measured on day 6 (Fig. 4B). In the case of A2780, co-culturing with fibroblasts at the C1:F2 ratio increased spheroid diameter compared to the co-cultures at the C1:F1 and C2:F2 ratios. For OV7, the spheroids formed at the C2:F1 ratio were larger than those formed at the C1:F1 ratio. No differences in diameter were observed between the co-culture ratios for the SKOV3 or COV362 cell lines.

By day 6, spheroid compactness was assessed across all conditions (Fig. 4C). In monoculture, A2780 exhibited low compactness, forming only cell aggregates. However, when co-cultured with patient-derived fibroblasts, the compactness increased at all ratios (C1:F1, C2:F1, C1:F2), indicating the formation of stable and well-defined spheroids. SKOV3 consistently formed compact spheroids under all conditions, and its compactness was unaffected by co-culturing with fibroblasts. COV362 displayed a ratio-dependent response: compactness decreased at the C2:F1 ratio but increased at C1:F2, with C1:F1 being comparable to monoculture. This suggests that a higher proportion of fibroblasts enhances spheroid compactness. OV7 showed greater compactness in co-culture, achieving the highest compactness at C1:F1 and a more modest increase at C1:F2. These observations demonstrate that fibroblast co-culture effects spheroid compactness in a manner that depends on both the specific cell line and the fibroblast-to-cancer cell ratio. Overall, the results show that increasing the proportion of fibroblasts enhances spheroid compactness in cell lines that typically produce less compact spheroids, while it does not affect the compactness in lines like SKOV3, which already form highly compact spheroids.

Gene expression was analyzed on day 6. For the cancer-dependent gene expression results, data were normalized to the 3D cancer monoculture (Fig. 4D). The A2780 cell line exhibited lower gene expression levels of *MMP9, SNAI1, POU5F1*, and *VEGF* across all co-culture ratios (C1:F1, C2:F1, C1:F2). In contrast, the SKOV3 cell line demonstrated higher expression of the *MMP9* and *SNAI1* genes. The COV362 cell line showed elevated gene expression for *MMP9, SNAI1*, and *VEGF*. Similarly, OV7 displayed increased levels of *SNAI1* and *VEGF*, along with a cell ratio-dependent expression of *MMP9*. The gene expression results from the four analyzed cancer cell lines in different culturing ratios indicate that in all lines capable of forming 3D cultures, an increase in *MMP9* and *SNAI1* gene expression was observed. Moreover, in the COV362 and OV7 cell lines, an increase in *VEGF* expression was noted; this increase is possibly linked to the greater spheroid compactness seen in these lines, as no *VEGF* increase was observed in the SKOV3 line, which already formed very compact 3D cultures. Conversely, the A2780 cell line displayed decreased expression of all analyzed genes in spheroid co-cultures.

Fibroblast dependent gene expression, data were normalized to fibroblast 3D monoculture (Fig. 4E). Co-cultures with SKOV3, COV362, and OV7 displayed higher expression of the *POU5F1* gene, although a cell ratio-dependent variation was noted. The OV7 cell line co-culture also exhibited increased expression of *SNAI1* and *VEGF* genes. Across all cell lines, a decrease in *FAP* gene expression was observed, which was used to define the amount of fibroblasts in the culture. The reduction in *FAP* gene expression suggests that the actively dividing cells responsible for spheroid size increase were the cancer cell lines used in the experiment.

### Fibroblast cell line WS1 enhance spheroid formation in ovarian cancer cell lines

**Fig. 5:**
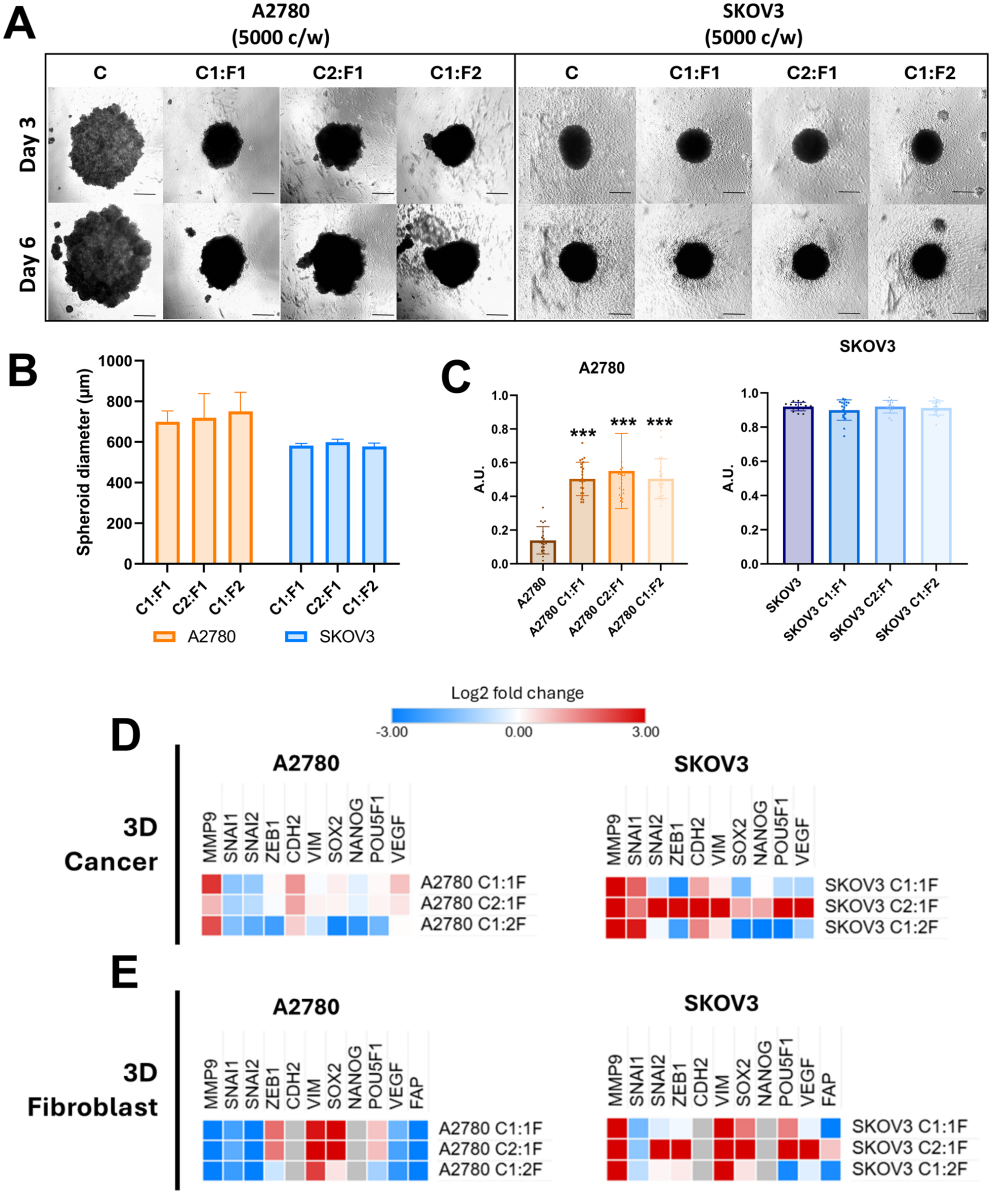
Fibroblast cell line co-culture modulates spheroid formation, size, compactness and gene expression in ovarian cancer cell lines. (**A**) Representative bright-field images of A2780, SKOV3 3D cultures co-cultured with fibroblast cell line WS1 fibroblasts at cancer: fibroblast ratios C1:F1 (1:1), C2:F1 (2:1) and C1:F2 (1:2) (total 5,000 cells/well (c/w)). Images are shown at day 3 and day 6. Scale bar represents 300 μm, magnification 40×. (**B**) Spheroid diameter at day 6. Bars show mean ± SD. *n* = 5–8, *N* = 3 biological replicates. (**C**) Spheroid compactness at day 6. A2780 shows low compactness in monoculture but increases at all co-culture ratios (C1:F1, C2:F1, C1:F2). Bars represent mean ± SD; *n* = 5–8, *N* = 3 biological replicates; points represent individual spheroids. ***- *p* < 0.001; statistical significance was assessed by unpaired Student’s t-test. (**D**) Heatmaps show log2 fold-change for *MMP9, SNAI1, SNAI2, ZEB1, CDH2, VIM, SOX2, NANOG, POU5F1, VEGF* normalized to each line’s 3D cancer monoculture. (**E**) Heatmaps show log2 fold-change for *MMP9, SNAI1, SNAI2, ZEB1, CDH2, VIM, SOX2, NANOG, POU5F1, VEGF FAP* normalized to 3D fibroblast monoculture

Human fibroblast cell line WS1 was used as a standardized stromal component to confirm fibroblast-driven effects on ovarian cancer 3D spheroid formation. WS1 fibroblasts were co-cultured with the ovarian cancer cell lines A2780 and SKOV3 (Fig. 5A). Co-cultures were prepared at cancer cell-to-fibroblast ratios of 1:1 (C1:F1), 2:1 (C2:F1), and 1:2 (C1:F2), with 5,000 total cells per well, and grown under 3D low-attachment conditions for 6 days. Representative brightfield images acquired on day 3 and day 6 demonstrate a clear, cell line-dependent response to WS1 co-culture (Fig. 5A). In monoculture, A2780 did not form compact spheroids and instead produced loose cell aggregates as seen previously in this study; however, when co-cultured with WS1 fibroblasts, A2780 developed well-defined, stable spheroids already by day 3 across all tested ratios, SKOV3 formed compact, round spheroids in monoculture and maintained this morphology in all co-culture conditions, with no obvious changes in overall spheroid shape or structural integrity across the different WS1 ratios.

Spheroid diameters were quantified on day 6 (Fig. 5B). While A2780 and SKOV3 spheroids showed consistent size ranges across the three co-culture ratios, no pronounced ratio-dependent differences in diameter were observed within either cell line, suggesting that WS1 primarily influenced spheroid organization rather than uniformly driving spheroid growth under these conditions. Spheroid compactness was assessed on day 6 (Fig. 5C). In monoculture, A2780 exhibited low compactness, consistent with aggregate formation; however, co-culture with WS1 significantly increased compactness at all ratios (C1:F1, C2:F1, and C1:F2) (*p* < 0.001), confirming the formation of stable and well-defined A2780 spheroids in the presence of fibroblasts. SKOV3 displayed high compactness in monoculture and remained comparably compact across all WS1 co-culture ratios, indicating that WS1 co-culture does not further enhance compactness in a cell line that already forms highly compact 3D spheroids.

Gene expression was analyzed on day 6 to evaluate cancer cell-dependent and fibroblast-dependent transcriptional responses. For cancer-dependent gene expression, data were normalized to the corresponding 3D cancer monoculture (Fig. 5D). In A2780 spheroids, WS1 co-culture was associated with increased expression of *MMP9* and a modest elevation of *CDH2* and *VEGF* genes, whereas *SNAI1* and *SNAI2* expression was downregulated across the different cell seeding ratios. In addition, stemness-associated genes (*SOX2, NANOG,* and *POU5F1*) were notably downregulated in the C1:F2 condition. In SKOV3 spheroids, WS1 co-culture resulted in higher expression of *MMP9, CDH2, VIM* and *SNAI1* at all ratios, and an increase in additional EMT/invasion-associated genes (*SNAI2, ZEB1, SOX2, NANOG, POU5F1*), with the most pronounced changes- together with increased *VEGF* expression- observed at the C2:F1 ratio. This indicates a ratio-dependent transcriptional response despite minimal morphological changes.

For fibroblast-dependent gene expression, data were normalized to WS1 3D monoculture (Fig. 5E). Across both co-cultures, a decrease in *FAP* expression was observed under all conditions except SKOV3 C2:F1, supporting a reduced relative fibroblast contribution within the mixed spheroids and suggesting that the actively dividing cells responsible for spheroid size were primarily the cancer cell fraction. WS1 gene expression responses were cell line–dependent: in A2780 co-cultures, *MMP9, SNAI1*, and *SNAI2* were downregulated, whereas *VIM* and *SOX2* (and to a lesser extent *POU5F1*) were upregulated. In SKOV3 co-cultures, WS1 displayed increased *MMP9* and *VIM* gene expression, accompanied by ratio-dependent modulation of *SNAI2, ZEB1, POU5F1*, and *VEGF*. Together, these data confirm that WS1 fibroblasts reproducibly modulate gene expression in a cancer cell line- and ratio-dependent manner, enhancing spheroid organization and compactness in A2780, while exerting limited effects on SKOV3 spheroid architecture but inducing a different transcriptional response.

### Printed micro-scaffold supports 3D culture formation

**Fig. 6:**
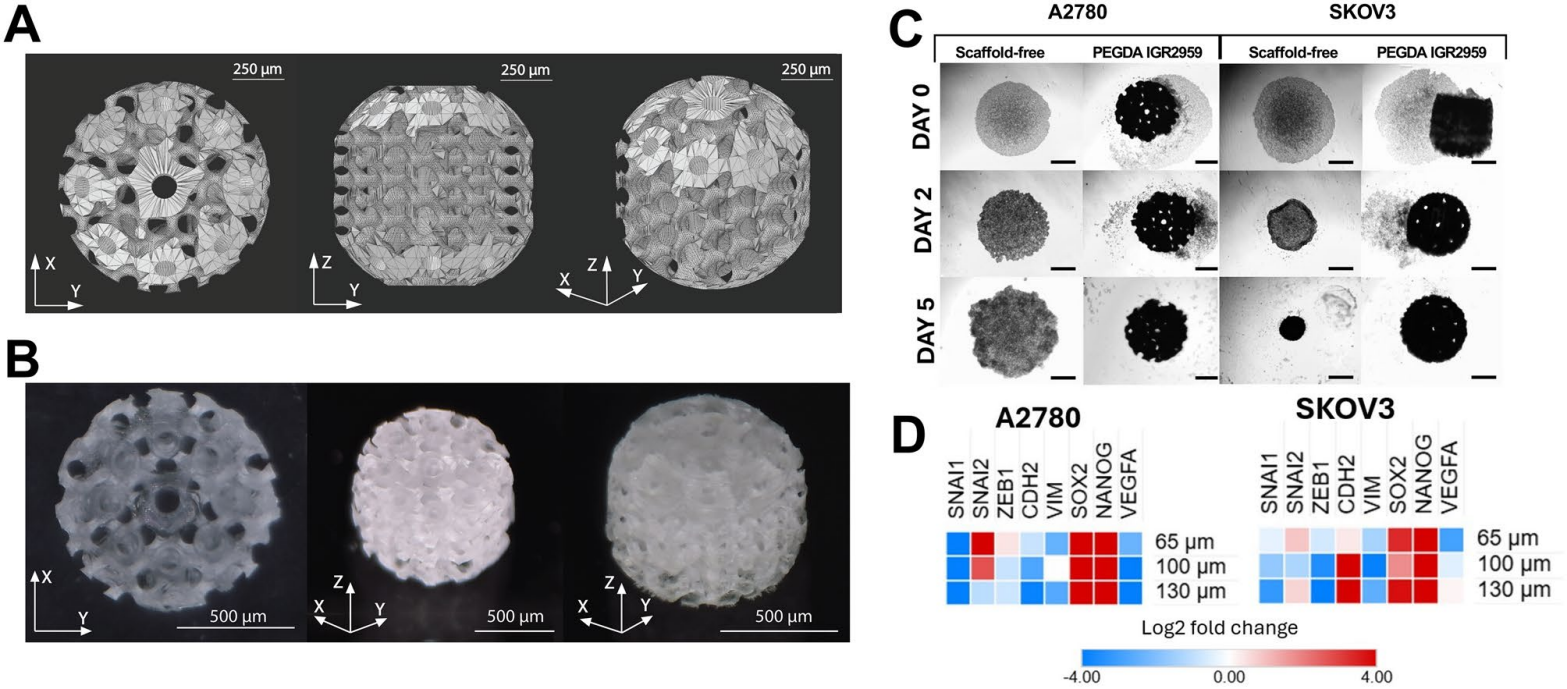
2PP produced PEGDA scaffolds support 3D cell culture formation. (**A**) Printed scaffold design (three orthogonal views) of the spherical/gyroid-like PEGDA scaffold showing circumferential and axial microchannels and through-pore architecture. Designs represent 100 µm pore size scaffold. Scale bars indicate 250 µm. (**B**) Optical microscope images confirming shape fidelity after printing: regular circular pores on the X–Y face, continuous channels along Z, and intact struts; microscale surface roughness is visible at pore rims and strut sidewalls. Scale bars as indicated 500 µm. Images represent 100 µm oriented scaffold. (**C**) Representative images of SKOV3 and A2780 seeded on scaffolds (14,000 cells/scaffold) and imaged at day 0, day 2, and day 5. Scale bars represent 300 µm. Images represent used 100 µm pore size scaffold in the culture. (**D**) Gene expression on day 6 presented as log2 fold-change relative to scaffold-free 3D monoculture. Heatmaps show *SNAI1, SNAI2, ZEB1, CDH2, VIM, SOX2, NANOG, VEGF* for each pore size (65, 100, 130 µm)

2PP printed PEGDA micro-scaffolds were fabricated with through-pore diameters of 65, 100, or 130 µm. The design renderings in three orthogonal views (Fig. 6A) show the intended spherical/gyroid-like architecture with circumferential and axial microchannels, providing continuous diffusion paths through the construct (overall scaffold diameter ~1 mm). Optical microscope images (Fig. 6B) confirm shape fidelity after printing: regular circular pores on the X–Y face, continuous channels along Z, and intact struts across the scaffold. Measured dimensional deviations from the Computer-Aided Design (CAD) model (Fig. 6A) were in the ~5 µm range (≈1% of overall size), consistent with material shrinkage during processing [26]. The PEGDA surface exhibits a microrough texture (~1 µm Root Mean Square roughness (RMS)), visible at the strut sidewalls and pore rims, which is advantageous for cell attachment [27] and also compatible with faster print times (5 µm slicing/hatching; 1 cm·s^− 1^ translation). The taller spherical constructs were produced by a submerged-vat, layer-lift approach to overcome objective working-distance limits; typical print time was ~1.5 h for one spherical scaffold as described previously [10]. The printed scaffolds were mechanically robust and easy to handle, with no post-release breakage observed. The mechanical properties of PEGDA were not characterized in this work. The main reason is that, due to the complex shape of the scaffolds, the use of atomic force microscopy (AFM) or other mechanical characterization techniques might not have yielded sufficiently accurate results. However, measurements performed in the literature on 3D PEGDA structures printed using a comparable 2PP setup showed that Young’s modulus, depending on the thickness and configuration of the object, can be in the range from 0.25 to 1.1 MPa [28]. Collectively, Figures 5A and B verify that the intended pore sizes and channel continuity were achieved and that the printed micro-architecture is suitable for subsequent cell culturing.

Cells were seeded onto PEGDA scaffolds with pore sizes of 65, 100, and 130 µm at a density of 14,000 cells per scaffold. The cells were imaged on day 0, day 2, and day 5. Two different cell lines were used: A2780, which does not form 3D cultures effectively, and SKOV3, known for forming well-structured and compact 3D cultures. As shown in Fig. 6C, by day 2, both cell lines had migrated closer to the scaffold and began to attach. By day 5, it was clear that all cells present in the well successfully attached to the scaffold. The absence of visible cell clumps or debris in the culture suggests that the cells adhered to the scaffold remained viable. Gene expression was analyzed on day 6 and normalized to the corresponding scaffold-free 3D monoculture, set to a value of 1 (Fig. 6D). In the SKOV3 cell line, a decrease in expression was observed for EMT-associated genes *SNAI1, ZEB1*, and *VIM*, while an increase was noted for *SNAI2, CDH2, SOX2*, and *NANOG.* The A2780 cell line exhibited an increase in the same genes observed in the SKOV3 cell line, such as *SNAI2, SOX2*, and *NANOG*. Interestingly, there was no increase in *VEGF*, a gene typically associated with higher expression in 3D cultures [11, 29], as also shown in this study (Fig. 3E; Fig. 4D; Fig. 5D). In fact, when comparing cells cultured under 3D conditions with a scaffold to those without, a decrease in *VEGF* expression was observed.

## Discussion

This study evaluates two complementary methods for creating reliable 3D cultures of ovarian cancer. We compared two distinct approaches: a scaffold-free method involving co-culture with patient-derived fibroblasts or fibroblast cell line WS1, and a scaffold-based method utilizing engineered and printed PEGDA micro-scaffolds. We used four ovarian cancer cell lines, A2780, SKOV3, COV362, and OV7, each exhibiting a unique molecular profile described in this study. Co-culturing with fibroblasts derived from ovarian cancer patient or fibroblast cell line induced significant cell line-specific responses. Notably, this approach resulted in the formation of compact 3D cultures in the A2780 cell line, which typically does not form spheroids under the standard culturing conditions used in this study. Increased compactness was also observed in the spheroids of the COV362 and OV7 cell lines co-cultured with CAFs. Furthermore we observed, scaffolds created using 2PP with PEGDA providing a stable structure, leading to the successful formation of 3D cultures in the A2780 cell line. Overall, these findings recommend a clear decision framework: utilize fibroblasts to investigate stromal-responsive heterogeneity and enhance spheroid compactness, while use printed scaffolds to handle variability and induce 3D-like cell states. As we observed in this study, printed scaffolds allow for more repeatable results regarding 3D cultures, while co-cultures involving patient-derived fibroblasts or fibroblast cell line addresses the heterogeneity caused by cell-to-cell interactions.

From a translational perspective, our paired platforms address two complementary knowledge gaps in ovarian cancer modelling: the frequent absence of stromal components in cancer cell-only spheroids, and the limited standardization of early 3D culture conditions that constrains reproducibility in preclinical testing. First, CAF or fibroblast co-culture provides a controllable way to introduce stromal signaling that is directly implicated in relapse and chemoresistance, enabling stromal-dependent phenotypes (e.g., compaction and EMT/angiogenesis-associated programs) to be measured in a line-specific manner [30, 31]. Consistent with this, recent ovarian cancer studies show that CAFs can protect tumor cultures from standard chemotherapies and shift drug-response programs, reinforcing the clinical relevance of including fibroblasts in preclinical testing [31, 32]. Second, architected PEGDA micro-scaffolds provide geometric control and a standardized starting microenvironment that helps reduce early aggregation variability while maintaining 3D-like transcriptional states, which is important for generating repeatable baseline readouts and for benchmarking drug responses across cancer cell lines. Notably, 2PP-fabricated scaffolds have been shown to support ovarian cancer 3D culture formation without additional coating and can drive measurable gene-expression shifts even in cell lines that are otherwise inconsistent in scaffold-free growing conditions [10]. Together, these two systems form a practical translational framework: CAF, fibroblast co-cultures are best suited to interrogate stromal-driven heterogeneity and resistance mechanisms, whereas printed scaffolds support repeatable, scalable 3D testing where controlling early variance is critical for robust preclinical drug screening and comparative studies.

In ovarian cancer, fibroblasts are abundant in solid tumor stroma, ascites and the omentum, actively remodeling the tumor environment by providing matrix components, cytokines, and chemokines. These factors influence cell aggregation, survival, and responses to therapy [33, 34]. Mechanistically, co-culture experiments accelerate the compaction of cells and can transform loose aggregates into spheroids. The order in which cells are seeded plays a critical role in determining the architecture of these spheroids; when seeding occurs simultaneously, fibroblasts infiltrate the core of the spheroid [12, 35]. This phenomenon mirrors observations in patient ascites, where heterotypic spheroids often show a core of CAFs encased by tumor cells expressing integrin-α5 [36]. These characteristics have also been replicated in scaffold-free ultra-low attachment system, revealing changes in proliferation and drug sensitivity compared to monoculture environments by Flörkemeier, I., et al. [12]. In the same study, similar to our findings that fibroblasts cause A2780 cells to transition from loose aggregates to compact spheroids, it was also reported that the addition of fibroblasts resulted in A2780 spheroid formation and them becoming smaller and more compact. In contrast, the OvCar8 cell line used in their study showed that spheroids became larger and more rounded at an earlier stage in coculture. Additionally, in coculture we observed increased compactness in other less compact COV362 and OV7 3D cultures. One notable difference between the studies is the source of the fibroblasts used; authors used dermal Detroit-551 fibroblasts or patient-matched peritoneal fibroblasts, while we utilized fibroblasts derived from ovarian tumor or WS1 fibroblast cell line. Furthermore, in our study fibroblasts enhanced the expression of genes associated with EMT and angiogenesis, including *MMP9, SNAI1*, and *VEGF* [37] in A2780, SKOV3, COV362, and OV7 3D cultures. This fibroblast-tumor interaction observed varies by cell line, making it particularly valuable for exploring stromal-responsive heterogeneity.

In comparison to patient-derived fibroblasts of fibroblast cell line as scaffold-free system, engineered PEGDA micro-scaffolds created through 2PP that allows for precise control over lattice geometry and the formation of continuous pore networks were used [38]. This standardization of printed scaffolds enhances early cell attachment and transport of nutrients in the media, leading to more consistent 3D gene expression readouts across various cell lines. In tumor models, 2PP-fabricated scaffolds have been shown to improve spheroid formation without the need for additional coating [10]. This aligns with our findings as 3D cultures consistently upregulated *SOX2* and *NANOG* genes without increasing *VEGF* levels compared to scaffold-free 3D cultures. Architected scaffolds have continuous pore networks that enhance oxygen and nutrient transport while standardizing local cell density. This likely reduces the upregulation of *VEGF* that is induced due to hypoxia stress in scaffold-free 3D environments [29]. Notably, brightfield imaging of compact scaffold-free spheroids revealed visually darker central regions, a feature commonly reported in association with hypoxic and/or necrotic core formation in larger tumor spheroids [13]. While hypoxia was not directly quantified in the present study, these morphological observations are consistent with diffusion-limited conditions in dense spheroids. In addition to enhancing formation efficiency, scaffold design serves as an experimental tool: researchers can adjust pore size, curvature, and strut spacing to influence diffusion pathways and mechanotransduction. Furthermore, the chemistry of PEGDA allows for surface functionalization with specific matrix cues, which are features that consistently highlight the advantages of 2PP over self-assembly methods alone [39].

When the research goal is to reveal stromal-responsive heterogeneity and define it or recreate stromal features, use scaffold-free co-culture with patient-derived fibroblasts or fibroblast cell line, we recommend adjusting the cancer cell : fibroblast ratio (e.g., 2:1, 1:1, 1:2), document the donor and passage, as fibroblast-tumor interactions vary by cell line and cell ratio, leading to increased variance between conditions. Ovarian cancer commonly exhibits immune cold/excluded states in which CAF-rich, TGF-β–dominant stroma and dense extracellular matrix (ECM) prevent T-cell infiltration [40, 41]. While our models do not include immune cells, CAF or fibroblast co-cultures may recapitulate stromal features of exclusion more than scaffold-only systems. Future addition of immune cells such as T cells or macrophages into CAF or fibroblast and cancer cell co-cultures could be an approach in recreating more precise tumor microenvironment. When the objective is to reduce early variability and achieve repeatable results while preserving 3D-like cell states, utilizing 2PP printed PEGDA micro-scaffolds could be a more feasible option. Specifying pore size, curvature, and strut spacing standardizes early cell attachment and diffusion. Consistent starting cell numbers and time points across different cell lines should be ensured too to reduce variability as well.

This study has several limitations that affect interpretation. Our co-culture experiments utilized a single patient-derived fibroblast donor at an early passage (P2) or fibroblast cell line WS1. While this choice limits our ability to assess variation due to different donors and passage-related differences, it minimized in vitro drift at the proof-of-concept stage. Additionally, cross-method comparisons were conducted at a single time point, using appropriate starting cell numbers and densities for each method. To enhance our findings, we examined multiple ovarian cancer cell lines (A2780, SKOV3, COV362, OV7) and tested different initial cell densities across complementary 3D systems, including fibroblast co-cultures and printed PEGDA micro-scaffolds. Together, these choices provide a focused and relevant observation of how stromal cues and engineered architecture influence 3D culture formation and gene expression.

Nevertheless, fibroblast co-culture combined with 2PP fabricated PEGDA micro-scaffolds provides complementary tools for 3D ovarian cancer modelling. Fibroblasts that are a very important component of tumor stroma and its formation can also enhance spheroid formation in vitro. In contrast, architected scaffolds help control early variability and produce consistent results while preserving 3D-specific characteristics and preventing *VEGF* elevation compared to scaffold-free 3D environments. Together, these results support a double strategy. Scaffold-free co-culture with patient-derived fibroblasts is best to reveal stromal-responsive heterogeneity and increase spheroid compactness in a line-specific manner, whereas 2PP printed PEGDA scaffolds provide architectural control that stabilizes early attachment and yields more repeatable 3D-like cell states. Importantly, both routes enabled A2780, a cell line that usually does not form spheroids, to form stable 3D cultures. Accordingly, model choice should follow the question: use co-culture to study cell–cell interactions and variance; use printed scaffolds to reduce early variability while preserving physiologically relevant architecture.

## Conclusions

To address the challenge that in vitro ovarian cancer models often need to balance biologically relevant heterogeneity with sufficient experimental control, we propose a double-strategy approach that utilizes both scaffold-free co-culture and architected scaffolds for 3D culture formation. In this study, we compared scaffold-free co-culture with patient-derived cancer-associated fibroblasts or fibroblast cell line WS1 and PEGDA micro-scaffolds fabricated by two-photon polymerization across four ovarian cancer cell lines with distinct molecular profiles. We identified that co-culture with patient-derived fibroblasts promotes more compact 3D cultures in a line- and ratio-dependent manner and enables A2780, which under standard conditions forms only loose aggregates, to establish stable spheroids while enhancing the expression of EMT- and angiogenesis-associated genes. Furthermore, we demonstrated that architected PEGDA micro-scaffolds with defined gyroid pore geometries provide a stable structure that supports reproducible 3D cultures in both spheroid-forming and non-spheroid-forming cell lines and shifts EMT/stemness-associated gene expression without excessive *VEGF* upregulation compared to scaffold-free 3D culture. Together, these findings outline a practical decision framework in which fibroblast co-culture is used to investigate stromal-responsive heterogeneity and cell–cell interactions, while 2PP-printed PEGDA micro-scaffolds are employed to reduce early variability and standardize 3D culture formation, suggesting a generalizable engineering paradigm for tailoring 3D ovarian cancer models to specific experimental aims.

## Electronic supplementary material

Below is the link to the electronic supplementary material.

Supplementary Material 1

## Data Availability

The datasets generated and/or analyzed during the current study are available from the corresponding author on reasonable request.
