# Supplementary material for: Balancing stromal-induced complexity in 3D ovarian cancer models through heterotypic co-culture with fibroblasts or architected micro-scaffolds
Source: J Biol Eng. 2026 Mar 30;20:87. doi: 10.1186/s13036-026-00670-9 (PMC13159347; doi:10.1186/s13036-026-00670-9)
Supplement: Supplementary file 1 — Supplementary Material 1 [file 13036_2026_670_MOESM1_ESM.docx]

**Supplementary Table 1**

**Table 1.** Gene Forward and Reverse Primers.

| **Gene** | **Forward Primer (5’-3’)** | **Reverse Primer (3’-5’)** |
| --- | --- | --- |
| ***ABCC1*** | CTCTATCTCTCCCGACATGACC | AGCAGACGATCCACAGCAAAA |
| ***ABCC5*** | AGTCCTGGGTATAGAAGTGTGAG | ATTCCAACGGTCGAGTTCTCC |
| ***ABCG2*** | CAGGTGGAGGCAAATCTTCGT | ACCCTGTTAATCCGTTCGTTTT |
| ***ATP11B*** | GACCCACCACATCAGAGTGAC | TGAGGTGTGTAAAGGCCATTCT |
| ***ATP7B*** | GCCAGCATTGCAGAAGGAAAG | TGATAAGTGATGACGGCCTCT |
| ***CD274*** | TGGCATTTGCTGAACGCATTT | TGCAGCCAGGTCTAATTGTTTT |
| ***CDH1*** | CGAGAGCTACACGTTCACGG | GGGTGTCGAGGGAAAAATAGG |
| ***CDH2*** | TCAGGCGTCTGTAGAGGCTT | ATGCACATCCTTCGATAAGACTG |
| ***CTNNB1*** | AAAGCGGCTGTTAGTCACTGG | CGAGTCATTGCATACTGTCCAT |
| ***FAP*** | ATGAGCTTCCTCGTCCAATTCA | AGACCACCAGAGAGCATATTTTG |
| ***FN1*** | CGGTGGCTGTCAGTCAAAG | AAACCTCGGCTTCCTCCATAA |
| ***IFNG*** | TCGGTAACTGACTTGAATGTCCA | TCGCTTCCCTGTTTTAGCTGC |
| ***MMP9*** | TGTACCGCTATGGTTACACTCG | GGCAGGGACAGTTGCTTCT |
| ***NACC1*** | CTGGCTCCTACCACAATGAGG | TGGCCGACGTTCATCATGC |
| ***NANOG*** | TTTGTGGGCCTGAAGAAAACT | AGGGCTGTCCTGAATAAGCAG |
| ***NOTCH3*** | TGGCGACCTCACTTACGACT | CACTGGCAGTTATAGGTGTTGAC |
| ***PCNA*** | CCTGCTGGGATATTAGCTCCA | CAGCGGTAGGTGTCGAAGC |
| ***POU5F1*** | CTGGGTTGATCCTCGGACCT | CCATCGGAGTTGCTCTCCA |
| ***RPL13a*** | GCCATCGTGGCTAAACAGGTA | GTTGGTGTTCATCCGCTTGC |
| ***SMAD2*** | CGTCCATCTTGCCATTCACG | CTCAAGCTCATCTAATCGTCCTG |
| ***SMAD3*** | TGGACGCAGGTTCTCCAAAC | CCGGCTCGCAGTAGGTAAC |
| ***SNAI1*** | TCGGAAGCCTAACTACAGCGA | AGATGAGCATTGGCAGCGAG |
| ***SNAI2*** | CGAACTGGACACACATACAGTG | CTGAGGATCTCTGGTTGTGGT |
| ***SOX2*** | GCCGAGTGGAAACTTTTGTCG | GGCAGCGTGTACTTATCCTTCT |
| ***TGFB1*** | GGCCAGATCCTGTCCAAGC | GTGGGTTTCCACCATTAGCAC |
| ***TGFBR1*** | ACGGCGTTACAGTGTTTCTG | GCACATACAAACGGCCTATCTC |
| ***TGFBR2*** | GTAGCTCTGATGAGTGCAATGAC | CAGATATGGCAACTCCCAGTG |
| ***TWIST1*** | GTCCGCAGTCTTACGAGGAG | GCTTGAGGGTCTGAATCTTGCT |
| ***VEGF*** | AGGGCAGAATCATCACGAAGT | AGGGTCTCGATTGGATGGCA |
| ***VIM*** | GACGCCATCAACACCGAGTT | CTTTGTCGTTGGTTAGCTGGT |
| ***ZEB1*** | GATGATGAATGCGAGTCAGATGC | ACAGCAGTGTCTTGTTGTTGT |

**Supplementary Figure S1**

**
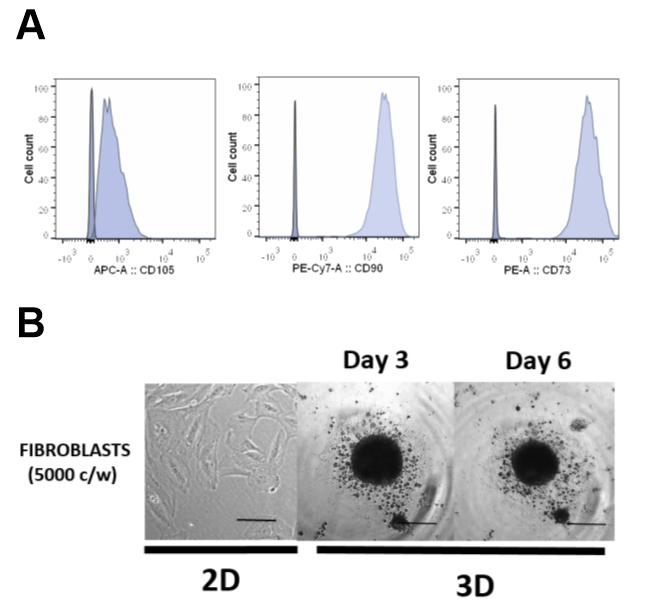
**

**Supplementary Fig. S1 Characterisation and 3D spheroid formation of patient-derived fibroblasts.**

(A) Flow cytometry analysis of fibroblasts isolated from ovarian tumour. Passage 2 cultures were stained for CD105, CD90 and CD73 (blue histograms); unstained controls are shown in dark blue. All three markers were strongly expressed.

(B) Images of fibroblasts cultured either in 2D or under 3D conditions. Fibroblasts were seeded at 5,000 cells/well (c/w); Scale bar represents 300 μm. Magnification 40x.
